# High tibial osteotomy in the ACL-deficient knee with medial compartment osteoarthritis

**DOI:** 10.1007/s10195-016-0413-z

**Published:** 2016-06-29

**Authors:** Benjamin V. Herman, J. Robert Giffin

**Affiliations:** Fowler Kennedy Sport Medicine Clinic, Western University, London, ON Canada

**Keywords:** Osteotomy, Chronic ACL deficiency, Varus gonarthrosis

## Abstract

High tibial osteotomy (HTO) has traditionally been used to treat varus gonarthrosis in younger, active patients. Varus malalignment increases the risk of progression of medial compartment osteoarthritis and an HTO can be performed to realign the mechanical axis of the lower limb towards the lateral compartment, thereby decreasing contact pressures in the medial compartment. Anterior cruciate ligament (ACL) insufficiency may lead to post-traumatic arthritis due to altered joint loading and associated injuries to the menisci and articular cartilage. Understanding the importance of posterior tibial slope and its role in sagittal knee stability has led to the development of biplane osteotomies designed to flatten the posterior tibial slope in the ACL deficient knee. Altering the alignment in both the sagittal and coronal planes helps improve stability as well as alter the load in the medial compartment. Detailed history, physical exam and radiographic analysis guide treatment decisions in this high demand patient population. Lateral closing wedge (LCW) and medial opening wedge (MOW) HTOs have been performed and their potential advantages and disadvantages have been well described. Given the triangular shape of the proximal tibia, it is imperative that the surgeon pay close attention to the geometry of the osteotomy “gap” when performing MOW HTO to avoid inadvertently increasing the posterior tibial slope. Simultaneous ACL reconstruction may require technique modifications depending on the type of HTO and ACL graft chosen. With appropriate patient selection and good surgical technique, it is reasonable to expect patients to return to activities of daily living and recreational sports without debilitating pain or instability.

## Introduction

High tibial osteotomy (HTO) has been used in the treatment of varus gonarthrosis since being popularized by Coventry in the 1960s [1]. Although ligament insufficiency was originally considered a contraindication to HTO, realignment surgery is now considered an important part of the treatment algorithm for the unstable knee. HTO realigns the mechanical axis of the lower limb and unloads the affected compartment, thereby transferring weight-bearing forces to the healthy knee compartment [2]. Furthermore, biomechanical studies have shown that planned alteration of the posterior tibial slope can also help improve or restore stability in the sagittal plane in ligament deficient knees [3]. Thus, valgus-producing HTOs, either lateral closing wedge (LCW) or medial opening wedge (MOW), should be considered as a preferred alternative to a knee arthroplasty, particularly in young, active patients with degenerative changes and concomitant cruciate injuries [4, 5].

ACL deficiency alters knee kinematics and may contribute to accelerated degenerative changes, particularly in the medial compartment, with subsequent loss or injury to the meniscus or articular cartilage [6, 7]. Osteotomy or realignment surgery can be used in this setting to treat both pain and instability by altering the posterior tibial slope, thereby changing the sagittal plane alignment [8, 9] in addition to the coronal alignment. This can be performed in conjunction with a ligament reconstruction in either a simultaneous or staged fashion [10–16].

This review will address the biomechanical rationale for an HTO and the relationship with varus malalignment, posterior tibial slope and ACL insufficiency. Patient selection and surgical planning will be reviewed, as well as specific surgical techniques. Finally, results in the literature will be examined.

## Biomechanical background

Varus malalignment has been defined as an angle of 3° between the mechanical axes of the femur and tibia [17], or where the weight-bearing axis of the lower limb passes medial to the tip of the medial tibial spine [18, 19]. Recent investigations have shown that varus malalignment is both a potential cause of medial compartment osteoarthritis and a significant factor in its progression [20].

Varus alignment creates a constant, static adduction moment at the knee resulting in increased loads in the medial compartment and tension on the lateral structures during stance [21]. During gait, there is an additional dynamic adduction moment at the knee during single leg stance. Further varus malalignment increases the loads/tension in the ACL [22, 23] and can attenuate other soft tissue structures, such as the posterolateral corner [24]. ACL deficiency has a further negative effect on the biomechanical environment. ACL deficiency results in altered gait biomechanics, including decreased knee flexion moments [25–27] and increased external knee adduction moments [28]. Both of these gait analysis parameters have been shown to be risk factors in the development and progression of osteoarthritis in the knee [29–31].

High tibial osteotomy can correct these pathologic biomechanical conditions by realigning the weight-bearing axis towards the lateral compartment articular cartilage and reducing contact pressures in the medial compartment. One study has shown that the peak contact pressures are 70 % higher in the lateral compared to the medial compartment [32] when the mechanical axis passes through the center of the knee. HTO also decreases the tensile forces on the lateral structures by almost 60 % [33]. Studies have shown that the adduction moment at the knee is decreased by as much as 38 % after an HTO [33–35]. Changes in neuromuscular control that further reduce the adduction moment have also been seen [36].

**Fig. 1:**
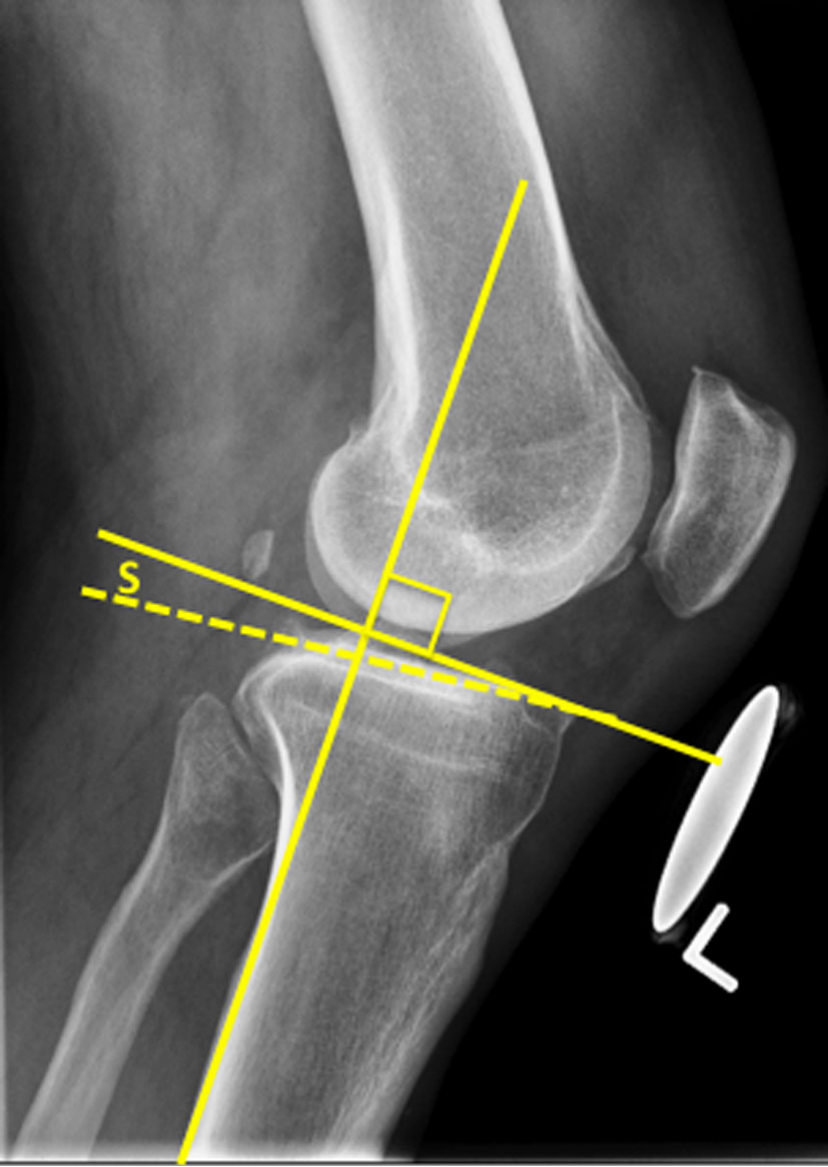
The measurement of posterior tibial slope (S)

The posterior tibial slope is defined as the angle between a line perpendicular to the mid-diaphysis of the tibia and the posterior inclination of the tibial plateaus (Fig. 1). On the medial plateau, this slope is usually 9–11°, whereas laterally it is typically 6–8° [37, 38]. This slope sets the biomechanical environment for the ACL and other structures, such as the posterior horn of the medial meniscus and lateral capsuloligamentous structures, to resist anterior tibial translation. Several studies have shown that increased slope causes anterior tibial translation [3, 39, 40]. Moreover, other studies have shown that increased slope increases the strain on the ACL [41, 42]. This increased strain may explain why patients with increased slope are at a higher risk for ACL injury [43–46]. With the apparent relationship between increased slope, ACL strain and anterior tibial translation, it is similarly theorized that decreasing the tibial slope will decrease ACL strain and anterior tibial translation, thus reducing instability symptoms [47, 48].

### Pre-operative assessment

Determining the suitability of a high tibial osteotomy for a patient with cruciate deficiency begins with a focused history and physical exam. On history, it is imperative to elicit the nature of the patient’s pain and instability. Patients complaining of symptoms of pain and “giving way” or instability during activities of daily living can be effectively treated with HTO alone, whereas patients complaining of both pain and instability primarily during pivoting sports are often better treated with realignment surgery in conjunction with a ligament reconstruction. It should be determined whether the patient experiences functional (giving way due to quadriceps inhibition or weakness) or true mechanical instability [8]. Identifying risk factors that can be modified or optimized (such as cessation of smoking or improved diabetic control) will improve patient selection and help decrease or avoid postoperative complications commonly associated with realignment surgery [49].

A focused physical exam begins with a gait assessment. The presence of a varus thrust is a strong indicator that osteotomy may be needed. Range of motion should be compared to the contralateral knee and any recurvatum or loss of flexion or extension should be noted. Ligamentous stability testing should be completed. It must be determined if there are other ligamentous injuries that need to be considered in a surgical plan. In a severe varus knee, there may be attenuation of the lateral and posterolateral corner structures that would elicit findings with varus stress, varus recurvatum or dial tests [50–53]. It is not uncommon to find pseudolaxity, or “correctable” varus, in patients with significant articular cartilage wear of the medial compartment. Lastly, a neurovascular exam and a peripheral vascular assessment should be done.

Standard radiographs should include bilateral weight-bearing anteroposterior (AP) views of the knee in full extension and in 30–45° of flexion (Rosenberg view), lateral, skyline and weight-bearing hip-to-ankle AP views [54]. The two AP views assess the joint space in extension and flexion. Posterior tibial slope and patellar height are assessed on the lateral view. Evidence of chronic ACL deficiency may be seen with increased posteromedial wear, also known as a “cupula” [55]. The standing hip-to-ankle view of both legs is used for an objective assessment of the deformity by measuring the mechanical axes.

**Fig. 2:**
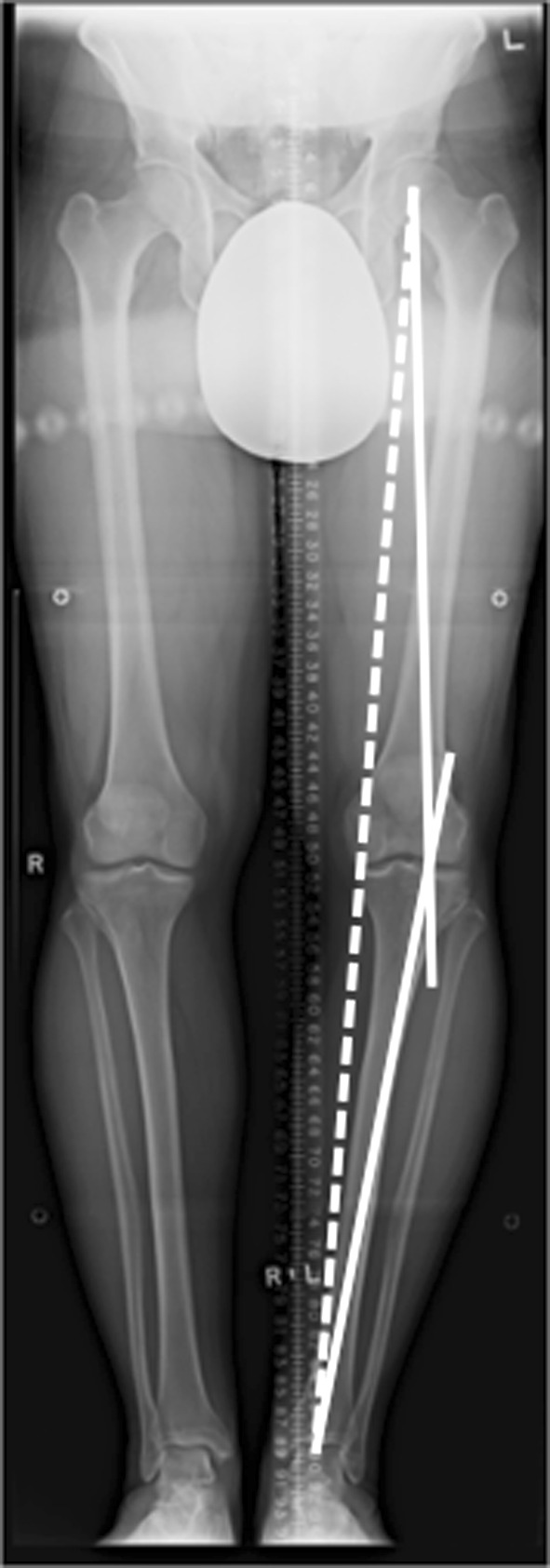
The two mechanical axes meet at the correction point, which is 62.5 % across the width of tibial plateau. The *dashed line* shows the weight-bearing axis falling through the middle of the medial compartment. The dashed line is the patient’s current weight-bearing axis

**Fig. 3:**
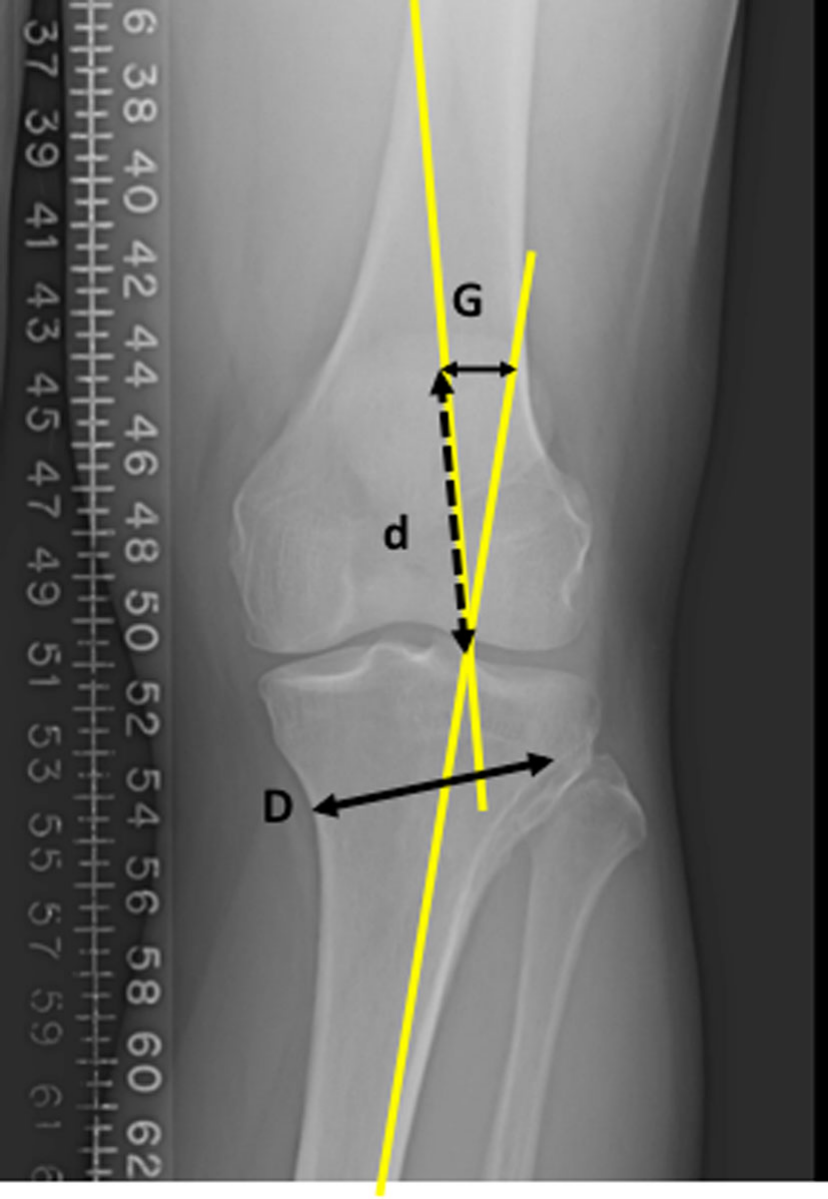
*Line D* shows the distance of the proposed osteotomy site and the same length is drawn over one of the mechanical axes. The distance between the two mechanical axes at this point (*line G*) provides what the size of the gap at the posterior aspect of the osteotomy should be in order to correct the weight-bearing axis to the correction point

The proposed osteotomy and size of correction is templated from the hip-to-ankle radiographs as well [54]. Typically, the new mechanical axis is repositioned to a point 62.5 % medial–lateral (medial edge = 0 %) across the tibial plateau [56], or can be roughly estimated as the midpoint of the downslope of the lateral tibial spine [18]. The mechanical axes of the femur and tibia are drawn through the correction point on the tibial plateau (Fig. 2). The osteotomy line is drawn on the proximal tibia, aiming for a point on the lateral metaphysis approximately 1–2 cm from the lateral joint line. The length of this line is measured, stopping about 5–10 mm short of the lateral cortex. This length is measured on one of the mechanical axes starting at the correction point. The distance between the two mechanical axes at the end of the overlaid line is the size of the gap needed to move the weight-bearing axis through the correction point (Fig. 3).

### Indications

Any patient considering realignment surgery must first maximize non-operative treatment options. Non-steroidal anti-inflammatory medications, injections, activity modifications, and weight loss should all be discussed initially. Trial of an unloader brace may be considered for patients wishing to avoid surgery. A course of physical therapy may improve a patient’s symptoms, and if not, will serve as “prehab” for a future surgery. Once these non-operative options have been exhausted, surgical options can be discussed.

Patients who are possible candidates for realignment surgery must have symptomatic (pain) varus malalignment. Patients with concurrent symptomatic instability are also candidates for tibial slope adjustment or a concurrent ACL reconstruction. HTO is best suited for physiologically young, high-level activity patients who wish to continue in their athletic endeavors or physically demanding jobs. More sedentary patients may be better suited for a total or partial knee arthroplasty, depending on the severity of their symptoms, degree of arthritis (i.e. end-stage), absence of deformity or tricompartmental disease. It may be necessary to perform an examination under anesthesia and a diagnostic arthroscopy before making the final treatment decision. Historically, the literature has suggested an upper age limit of 65 years to determine a patient’s suitability for HTO [57, 58]. However, the use of age alone as an exclusion criterion should be approached with caution. Ultimately, the older a patient is, the more ideal candidate they should be for HTO to be considered a viable alternative to a total knee arthroplasty. In younger patients with significant deformity requiring large corrections, HTO may be a bridge procedure that protects their knee until they reach an age when a total knee arthroplasty is considered to be more acceptable. Lastly, in patients with very early disease, HTO may be a disease modifying operation to avoid progression of osteoarthritis. With regards to ACL deficiency and varus alignment, there is evidence to suggest that the posterior tibial slope should be decreased when it is greater than 13° and associated chronic ACL deficiency evidenced by increased anterior tibial translation of at least 10 mm compared to the contralateral knee [48]. Slope and varus malalignment must also be scrutinized in younger patients who have had multiple failures of ACL reconstruction [25, 59]. The decision to perform an ACL reconstruction must be made based on the patient’s age and athletic pursuits. This can be staged, with the osteotomy performed first, or concurrently, depending on the surgeon’s preference [12, 14, 15, 37].

## Technique

Once the wedge size has been determined, the correction can be obtained via a medial opening wedge (MOW) or a lateral closing wedge (LCW) HTO. Both methods have been used for osteotomy with ACL deficiency [10–12, 14–16, 18, 37, 60]. Each technique has unique advantages and disadvantages.

The LCW HTO was the first method described [11], and provides immediate cortical contact which has traditionally allowed patients to fully weight bear earlier without the need for bone grafting. The LCW HTO has a tendency to decrease posterior tibial slope, which is advantageous in the ACL deficient knee [61, 62]. However, this method decreases proximal tibial bone stock, which may make a subsequent total knee arthroplasty technically more demanding [63]. The approach is also in close proximity to the common peroneal nerve, anterior compartment and proximal tibiofibular joint [9]. Injuries to these structures could result in iatrogenic nerve injury, compartment syndrome, and proximal tibiofibular joint instability.

The MOW HTO allows easier correction of coronal and sagittal alignment since one cut is easier to titrate than two parallel cuts [59]. This method preserves bone stock and may help “tighten” the capsuloligamentous structures around the knee [59], while avoiding the neurovascular risks of the LCW HTO. Disadvantages of the MOW include the possible need for bone graft and the risk of delayed or non union. Additionally, the potential to increase posterior tibial slope exists with an inexperienced surgeon or with lack of attention to detail. The absolute correction of tibial slope may be limited with MOW HTO by the triangular geometry of the tibia [64]. Nonetheless, the advantages of MOW make it the current preferred technique to address varus gonarthrosis in the ACL-deficient patient [8].

### MOW HTO surgical technique

A general anesthetic is preferred as the use of a regional block could mask potential post-operative compartment syndrome. The patient is positioned supine on a radiolucent operating table with a tourniquet applied to the upper thigh. A bolster may be placed under the ipsilateral hip to prevent excessive external hip rotation. Pre-operative antibiotics are administered. A sterile positioning bundle can be used underneath the knee and leg during the osteotomy to optimize positioning during fluoroscopy. The positioning bundle can also be used to place under the heel to extend the knee prior to final fixation. If knee arthroscopy is indicated, this is performed first.

Appropriately draped C-arm fluoroscopy should be brought in from the same side as the operative leg while the surgeon stands on the opposite side. This position allows easier access to the surgical wound on the medial side of the leg.

A medial longitudinal incision is made midway between the tibial tubercle and the posteromedial border of the tibia. Sartorial fascia is incised above the gracilis tendon. Fascial attachments posterior to the MCL at the metaphyseal flare of the posteromedial aspect of the tibia are elevated. Blunt dissection across the posterior cortex of the tibia elevates soft tissue and aids in placing a blunt tipped Hohmann retractor to protect neurovascular structures. This instrument will be in direct line with the proposed osteotomy. Anteriorly, the tibial tubercle and the medial border of the patellar tendon are identified and the fascia along the medial aspect is opened with cautery. This facilitates insertion of a blunt retractor (bent Lane or Hohmann) beneath the tendon.

A break-away guide pin is drilled in at the superior margin of the planned osteotomy site under fluoroscopic guidance. The tibial width can be measured using the calibrated pin. The pin should be directed towards a point at least 1 cm distal to the joint line on the lateral cortex. Ensure that the proposed osteotomy passes superior to the tibial tubercle. If the patellar tendon insertion is too close to the proposed osteotomy, a proximally directed biplane osteotomy that will allow the level of the tibial osteotomy to be lowered without compromising the extensor mechanism can be made posterior to the tubercle in the tibial tubercle. A biplanar osteotomy should also be performed for corrections greater than 12.5 mm to avoid significantly decreasing the patellar height [9, 65]. The biplane cut has the added advantage of increasing rotational stability of the osteotomy. In the sagittal plane the cut should be parallel to the tibial slope. In the case of an abnormally flat slope, the cut can angle back approximately 10°, which will provide more proximal bone for subsequent screw fixation. The planned osteotomy plane is marked with cautery. The superficial medial collateral ligament can be released with cautery in line with the proposed osteotomy. Alternatively, the superficial MCL may be elevated distal to the osteotomy and allowed to cover over the osteotomy and plate at the end of the procedure. If the superficial MCL is not released in some way, a medial tension band is created across the medial compartment, increasing compartment pressures [32].

Blunt retractors should remain in place both posteriorly and anteriorly when making the bony cut. A small oscillating saw blade 45 mm in length is chosen and the cut is made below and in line with the guide-pin. The direction of the saw blade should be confirmed with fluoroscopy to ensure it does not drift towards the knee joint. Thin osteotomes are used to complete the cut. It is advised that a bone bridge of approximately 10 mm be maintained on the lateral side and that the lateral extent of the osteotomy be closer to the lateral cortex than to the lateral joint line. This will help avoid a lateral cortical breach as well as intra-articular fracture propagation. Once it appears that the osteotomy has been completed anteriorly and posteriorly and is far enough lateral, a wide, firm osteotome can be inserted to assess its mobility. The osteotomy is gradually opened to the desired wedge correction as measured by the gap at the posteromedial aspect of the osteotomy site. This can be done with stacked osteotomes or wedges, and a laminar spreader may be required to maintain the wedge correction.

The sagittal alignment and tibial slope are assessed with a lateral image and compared to pre-operative imaging. In the absence of cruciate deficiency, the slope is kept neutral by maintaining a gap in the osteotomy site posteriorly that is twice as big as the gap anteriorly [64]. In the case of ACL deficiency, closing the gap anteriorly flattens the tibial slope. A 2° change in slope can be expected for every 1 mm of closure anteriorly [64]. This anteroposterior relationship of the osteotomy gap is due to the axial triangular geometry of the proximal tibia. As mentioned, closure of the anterior aspect of the gap can be achieved by placing a positioning bundle under the heel with the knee in extension during plate fixation.

With the osteotomy complete and the size of the gap and angle of the slope confirmed, the desired hardware can be applied. A locking plate system is preferred to maintain axial and rotational stability during the consolidation period. Screws are inserted under fluoroscopic guidance to ensure safe positioning. Once fixation is complete and hardware position is confirmed with fluoroscopy, the defect (i.e. gap >10 mm) can be filled with bone graft or a bone graft substitute. The wound is closed over a drain followed by the application of dressings and a hinged knee brace.

### Considerations when performing concurrent ACL reconstruction

For patients who are candidates for a concurrent ACL reconstruction, it must be ensured that the HTO hardware does not interfere with the tibial tunnel or fixation. The procedure should begin with arthroscopic assessment and treatment of concurrent meniscal and articular cartilage injuries. The osteotomy is then completed with slope either maintained or flattened at the discretion of the surgeon. Proximal screw fixation of the plate will have to be manipulated to avoid interference with the tibial tunnel. This can be accomplished by placing the plate as posterior as possible, with or without leaving the most anterior screw hole of the proximal aspect of the plate empty or short. If a screw hole is left empty, the surgeon must ensure the plating system allows for adequate fixation in the proximal fragment. Once the plate is secured to the bone, the ACL reconstruction is resumed including graft preparation and the drilling of both the femoral and tibial tunnels. The tunnel should exit the anterior tibial cortex at the proximal aspect of the osteotomy site. Tibial fixation of the ligament is at the surgeon’s discretion.

## Results

Good results have long been reported in HTO for varus malalignment alone [1, 2, 4, 5], and therefore the focus of this section will be results of HTO in ACL-deficient patients. Fowler et al. [60] reported on a small group of varus malaligned patients with chronic ACL-deficiency who underwent a LCW HTO alone. Good functional results were found in all patients. Noyes et al. [10] reviewed 41 patients with LCW HTO for symptomatic varus malalignment with a chronic ACL deficiency. Sixteen of the patients required a second stage ACL reconstruction; 78 % felt subjectively improved and 88 % would have the HTO again. The authors suggest that modifications limiting high-level athletics may be best. Naudie et al. [59] reported on 17 patients who received a MOW HTO for chronic ligamentous deficiencies. Five of the patients had ACL injuries as part of a chronic multiligamentous injury. All patients had improved knee stability, and all but one (not an ACL deficient patient) were satisfied with the osteotomy surgery.

Two studies have compared results of different treatment approaches to a varus, ACL-deficient patient. Latterman et al. [14] had 30 patients with 11 undergoing HTO alone, eight undergoing simultaneous HTO-ACL reconstruction, and eight a staged ACL reconstruction. LCW and MOW HTOs were both used. At an average follow-up of 5.8 years, pain with light activities was reported by one of 11 HTO patients, three of the eight simultaneous ACL reconstruction patients and four of the eight staged ACL reconstruction patients. A positive pivot shift was present in two of 11 in the HTO group, four in the simultaneous ACL group and three in the staged ACL group. Radiographic analysis showed progression of osteoarthritis in all patients, with no significant difference between the groups. Badhe et al. [15] reported on 14 patients (five with double varus syndrome and nine with triple varus syndrome) at 2.8 years of follow-up on average. These patients underwent a combination of LCW and MOW HTO. The double varus patients received a simultaneous LCW HTO and ACL reconstruction. Three of the nine triple varus patients received HTO alone and six had a simultaneous HTO with ligamentous procedure. Eighty-six percent of all patients were stable at last follow-up and 93 % were able to perform light recreational activities. Triple varus patients who had MOW HTO had better patient-reported outcomes than those who underwent LCW HTO.

Dejour et al. [12] reported on 44 patients who received simultaneous HTO and ACL reconstruction with both LCW and MOW HTO. Of these, 91 % were satisfied or very satisfied and 89 % had a negative or grade 1 Lachman test. Bonin et al. [37] reported on 30 patients who underwent simultaneous HTO and ACL reconstruction with both LCW and MOW HTOs. At a 12-year follow-up, 84 % of patients had gone on to play moderate or intense sports and only 17 % had radiographic progression of osteoarthritis greater than grade one. Noyes et al. [11] published results on 41 patients with double or triple varus syndrome. All initially had a LCW HTO, while 34 received a staged ACL reconstruction and 18 required a posterolateral corner reconstruction. Patient results were analyzed together and 71 % had decreased pain and 85 % had increased stability. Cincinnati knee scores improved from 63 to 82.

Lastly, Marriott et al. [16] performed gait analysis on 33 patients with varus malalignment, medial osteoarthritis (Kellgren–Lawrence grade I–III) and chronic ACL deficiency before and after they received a simultaneous MOW HTO and ACL reconstruction. Analysis was performed 2 and 5 years post-operatively, and compared to pre-operative measurements. The 5-year data showed a significant decrease in the peak knee adduction and flexion moments. These improvements in gait biomechanics were coupled with statistically significant improvements in Knee Injury and Osteoarthritis Outcome Scores. The authors concluded that a simultaneous HTO and ACL reconstruction is biomechanically efficacious procedure.

## Conclusion

High tibial osteotomy is an effective procedure in the surgical management of symptomatic, varus malaligned patients with an ACL deficiency. Decreasing posterior tibial slope has the ability to improve stability with or without the need for a ligament construction. It is important to note that as evidenced by the literature, there is a great deal of heterogeneity in this patient population, and that no simple algorithm exists for treatment. Nonetheless, with appropriate selection, patients can reasonably expect to perform activities of daily living and recreational sports without debilitating pain or instability.
